# Systemic lupus erythematosus gastrointestinal involvement: a computed tomography-based assessment

**DOI:** 10.1038/s41598-020-63476-9

**Published:** 2020-04-14

**Authors:** Zhiwei Chen, Jiaxin Zhou, Jiaoyu Li, Yiquan Zhou, Xiaodong Wang, Ting Li, Liyang Gu, Fangfang Sun, Wanlong Wu, Wenwen Xu, Shuhui Sun, Jie Chen, Jiajie Li, Liangjing Lu, Wen Zhang, Yan Zhao, Shuang Ye

**Affiliations:** 1 Department of Rheumatology, Renji Hospital South Campus, Shanghai Jiaotong University School of Medicine, Shanghai, 201112 China; 2 Department of Rheumatology, Peking Union Medical College Hospital, Peking Union Medical College and Chinese Academy of Medical Sciences, Key Laboratory of Rheumatology and Clinical Immunology, Ministry of Education, Beijing, 100730 China; 3 3Department of Clinical Nutrition, Renji Hospital, Shanghai Jiaotong University School of Medicine, Shanghai, 200127 China

**Keywords:** Computed tomography, Systemic lupus erythematosus

## Abstract

Systemic lupus erythematosus (SLE) gastrointestinal (GI) complication is characterized by multi-segment and multi-compartment involvement. The aim of this study is to develop a computed tomography (CT) image-based system for disease evaluation. SLE patients with GI involvement from two independent cohorts were retrospectively included. Baseline abdominal CT scan with intravenous and oral contrast was obtained from each individual. A CT scoring system incorporating the extent of GI tract involvement and intestinal wall thickness, along with extra-GI compartment involvement, was developed and validated. The outcome measurement was the time to GI functional recovery, defined as the time to tolerable *per os* (PO) intake ≥50% of ideal calories (PO50). A total of 54 and 37 patients with SLE GI involvement were enrolled in the derivation and validation cohorts, respectively. The CT scores for SLE GI involvement were positively correlated with patients’ time to PO50 (r = 0.57, p < 0.0001, derivation cohort; r = 0.42, p = 0.0093, validation cohort). Patients with a CT score ≤ 3 had a shorter time to PO50 (median time of 0 day) in pooled cohort, whereas those with a CT score > 3 incurred a significantly prolonged recovery with a median time to PO50 of 13 days (p < 0.0001). The CT-based scoring system may facilitate more accurate assessment and individualized management of SLE patients with GI involvement.

## introduction

Systemic lupus erythematosus (SLE) is a prototypic autoimmune disease with multi-system involvement^1^. Gastrointestinal (GI) manifestations are commonly presented in up to 50% of SLE patients^2^, with 2–30% of these cases attributed to active SLE *per se*^3^; whereas others may due to treatment side effects or co-morbidities.

Several key questions remain undetermined concerning active SLE GI involvement. First, multiplicity of its terminology, such as GI vasculitis (mesenteric vasculitis), lupus gastroenteritis (lupus enteritis) and intestinal pseudo-obstruction (IPO)^4–7^, implies the uncertainty of the underlying pathophysiology. Second, specific assessment tools for its activity, severity and outcome are lacking. As an example, none of the clinical features of SLE GI involvement is captured by the widely used SLE disease activity index (SLEDAI). It is noteworthy that computed tomography (CT) imaging has been well accepted in the evaluation of SLE GI involvement. Certain characteristic radiographic features^8,9^, such as ‘target sign’ (intestinal wall edema and thickening), ‘comb sign’ (enhanced engorgement of mesenteric vasculature), extra-GI compartment involvement (gallbladder wall thickening, interstitial cystitis, dilatation of urinary tract and biliary-pancreatic duct), have been described. However, image-based, prognosis-relevant measurable tools are yet to be developed. Third, the current treatment, particularly nutritional protocol for SLE patients with GI involvement, are merely empirical. Although the overall outcome is relatively benign^10^, some patients may suffer from a prolonged GI insufficiency, and some may have refractory or recurrent disease^9,11,12^. Evidence-based guidelines are unavailable to curb the disease management.

In the current study, we are aiming to focus on the second question, i.e., to develop a CT image-based evaluation system for SLE GI involvement in order to more precisely measuring the extent and severity of the disease, which hopefully, might also pave the path leading to better understanding and controlling this disease.

## Results

A total of 54 eligible SLE GI involvement cases were analyzed in the derivation cohort. The distribution of clinical manifestations is presented in Table 1, with abdominal pain (90.7%), nausea and vomiting (87.0%), and diarrhea (61.1%) being the most frequent symptoms. The median SLEDAI score was 7. Only one patient died due to myelodysplastic syndrome at the 3-month follow-up period. Comparable clinical characteristics can be found in the validation cohort (n = 37) (Table 1).

**Table 1:** Clinical characteristics, treatments, and outcomes.

	Derivation cohort (n = 54)	Validation cohort (n = 37)	p value
Age, y	34 (26.8–41.3)	30 (25–39.3)	0.095
Female, n(%)	52 (96.3)	35 (94.6)	0.99
SLE disease duration, month	30 (10.5–43.5)	11 (1.5–81)	0.38
Duration of GI symptoms to CT, week	2 (0.5–4)	5 (3–9)	<0.01
SLEDAI score	7 (5–12)	6 (3.5–13.5)	0.55
*Clinical manifestations*			
Abdominal pain, n(%)	49 (90.7)	32 (86.5)	0.73
Nausea and vomiting, n(%)	47 (87.0)	28 (75.7)	0.17
Diarrhea, n(%)	33 (61.1)	23 (62.2)	0.99
Fever, n(%)	11 (20.4)	10 (27.0)	0.46
Urinary tract symptoms, n(%)	7 (13.0)	5 (13.5)	0.99
Hematochezia, n(%)	3 (5.6)	3 (8.1)	0.68
Active lupus nephritis, n(%)	21 (38.9)	14 (37.8)	0.99
NPSLE, n(%)	2 (3.7)	4 (10.8)	0.21
*Treatments*			
Dosage of GC > 2 mg/kg/d, n(%)	33 (61.1)	21 (56.8)	0.83
Hydroxychloroquine, n(%)	30 (55.6)	27 (73.0)	0.12
Cyclophosphamide, n(%)	25 (56.8)	31 (83.8)	0.015
Mycophenolate mofetil, n(%)	11 (20.4)	5 (12.8)	0.57
Cyclosporine, n(%)	2 (3.7)	1 (8.5)	0.99
Azathioprine, n(%)	2 (3.3)	0 (0)	0.51
Rituximab, n(%)	4 (6.7)	0 (0)	0.14
TPN, n(%)	41 (75.9)	21 (56.8)	0.068
*Outcomes*			
3-months mortality, n(%)	1 (1.11)	0 (0)	0.99

Data are presented as median with interquartile ranges (Q1-Q3) if the distribution was skewed and otherwise expressed as mean ± SD for continuous variables and number (frequency) (%) for categorical variables.

NPSLE, neuropsychiatric SLE; SLEDAI, SLE disease activity index; GC, glucocorticoid expressed as prednisone dosage; TPN, total parenteral nutrition.

### Development of a CT image-based scoring system

According to previous reports, intestinal wall thickening was defined as at least 3-mm thickening in an area where the bowel was adequately distended^13,14^. To verify the accuracy of this intestinal thickness cutoff in SLE patients and the consistency of measurements by different CT investigators, SLE patients with gastrointestinal symptoms attributed to other causes (met exclusion criteria) were treated as control group (n = 42) (Supplementary material). Our data confirmed that 3 mm as a cutoff for intestinal thickening yielded good sensitivity (85.7%) and specificity (92.6%) for SLE GI involvement. The readout consistency between two trained investigators was fair (Kappa = 0.72, p < 0.001).

**Figure 1:**
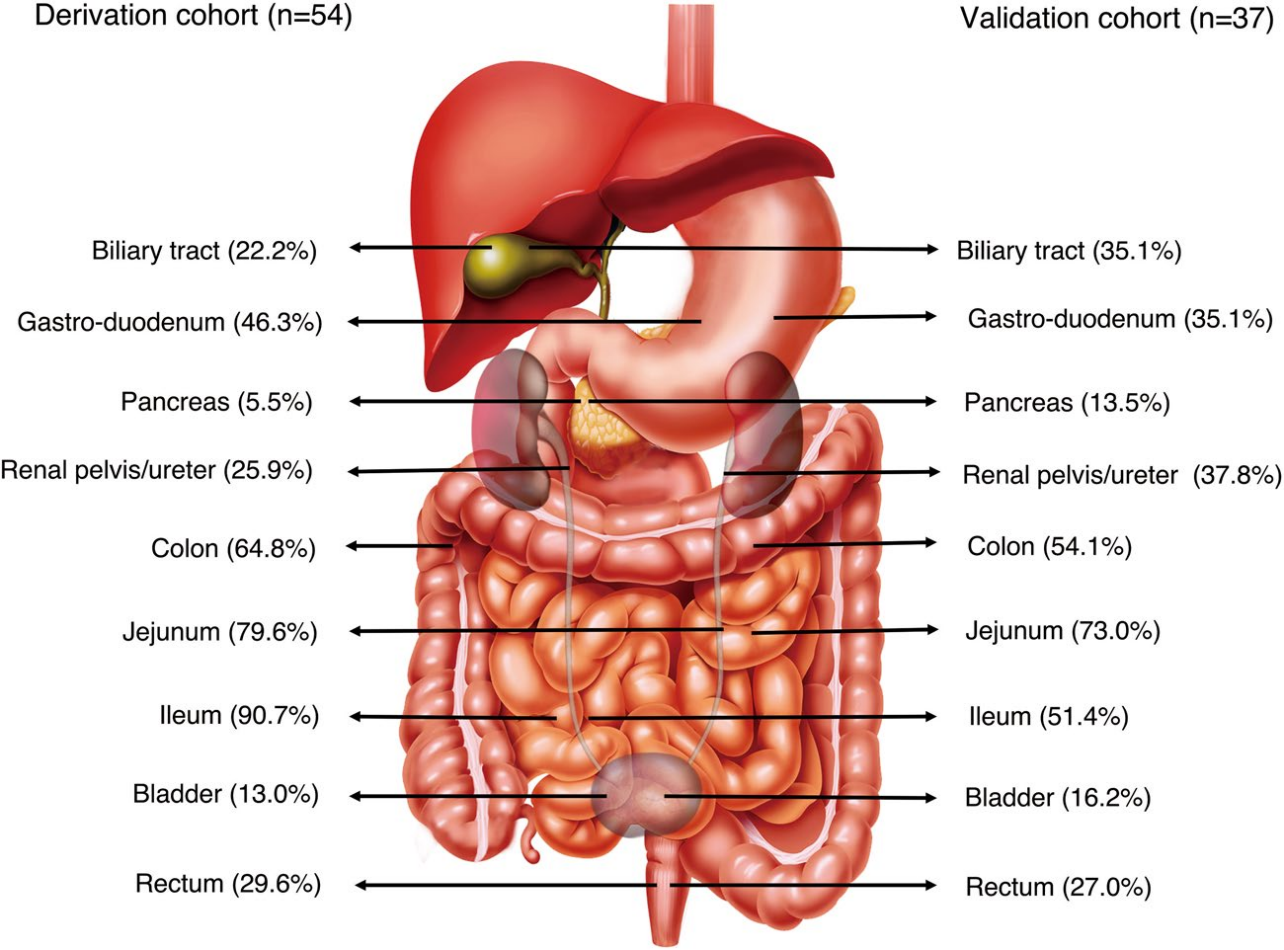
Anatomical distribution of SLE-VPO involvements (Part of the data was presented as a poster in Lupus Conference 2019^32^).

Subsequently, a descriptive analysis was carried out in the derivation cohort. 92.6% of the patients had GI tract thickening, and the most frequent segments of the GI tract affected were the ileum (90.7%), jejunum (79.6%), colon (64.8%), and gastro-duodenum (46.3%). Of note, 42.6% of patients had extra-GI compartment involvement, with ureterohydronephrosis (UH) as the most common feature (25.9%), followed by gall bladder/biliary duct involvement (22.2%). As comparison, in the validation cohort, the frequencies of affected GI tract were slightly lower, in the order of jejunum (73.0%), colon (54.1%), ileum (51.4%), and gastro-duodenum (35.1%), whereas the extra-GI involvements were more frequently presented, such as UH (37.8%) and gall bladder/biliary duct involvement (35.1%) (Fig. 1).

**Figure 2:**
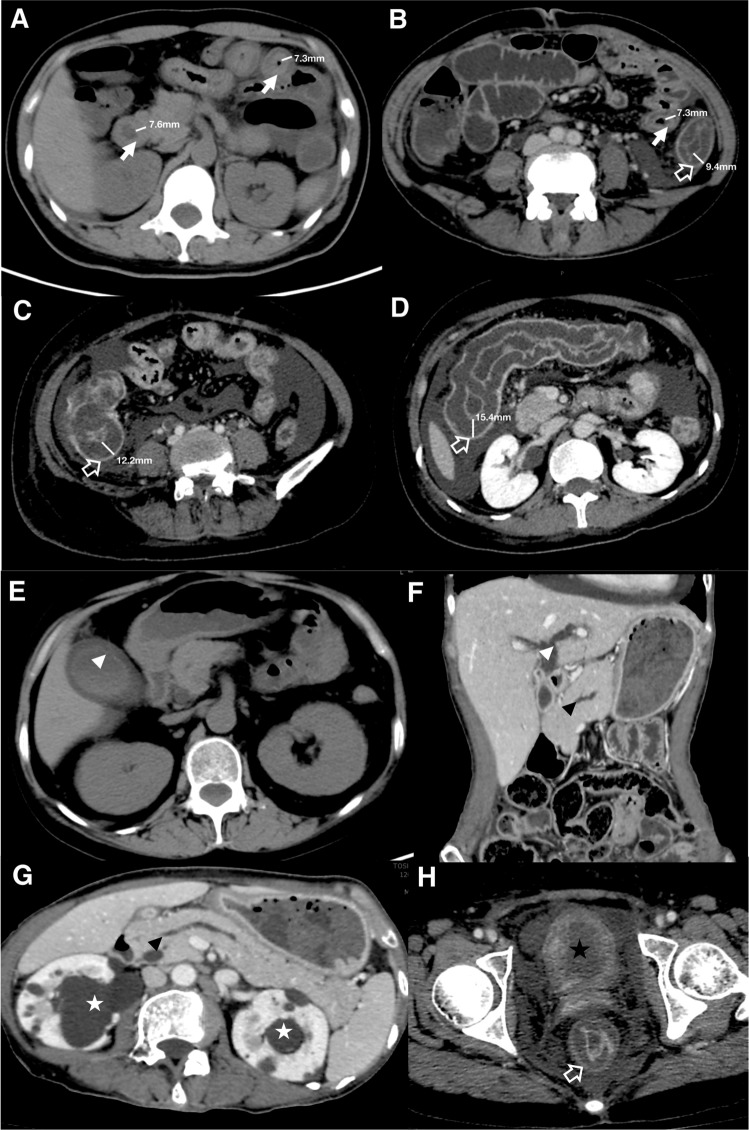
Illustrations of CT scoring. Representative images from 4 patients to illustrate CT scoring process (**A**–**H**). “White arrow” refers to small intestine (duodenum and jejunum, **A**; ileum, **B**). “White hollow arrow” refers to large intestine (descending colon, **B**; ileocecum, **C**; transverse colon, **D**; rectum, **H**). Measurements of the thickness of bowel walls are indicated by white bars. “Arrow head” refers to pancreatico-biliary system involvement (white arrow head: gall bladder wall thickening, **E**, biliary duct dilatation, **F**; black arrow head: pancreatic duct dilatation, **F**,**G**). “Asterisk” refers to urinary involvement (white asterisk: ureterohydronephrosis, **D**,**G**; black asterisk: bladder wall thickening, **H**).

As the surrogate of time to GI function recovery, the time to *per os* (PO) intake ≥50% ideal calories (PO50) served as the outcome measurement. The intestinal wall thickness and its anatomic location, along with extra-GI compartments involvement were all recorded. The itinerary to generate a composite model with robust correlation to PO50 can be found in Supplementary Table 1. Briefly, a CT image-based scoring system was developed combining the weighted thickness of 4 GI segments (duodenum, jejunum, ileum and colon) and the involvement of 4 extra-GI compartments (gallbladder/biliary tract, pancreas/pancreatic duct, renal pelvis/ureter, and bladder) (Table 2 and Fig. 2). The measurements of gastric and rectal wall thickness were removed due to their content-related high variability.

**Table 2:** CT scoring system.

Anatomical sites affected	Score (total score* = 12)
Thickness of bowel walls ≤3.0 mm, 0; 3.1–7.9 mm, 1; ≥8.0 mm, 2	
Duodenum	0/1/2
Jejunum	0/1/2
Ileum	0/1/2
Colon	0/1/2
Extra-GI	
Biliary tract (gallbladder/biliary duct)	0/1
Pancreas/pancreatic duct	0/1
Renal pelvis/ureter	0/1
Bladder	0/1

*The total CT score is determined by adding the maximum weight (score) in each item.

### CT scores predict SLE GI functional outcome

**Figure 3:**
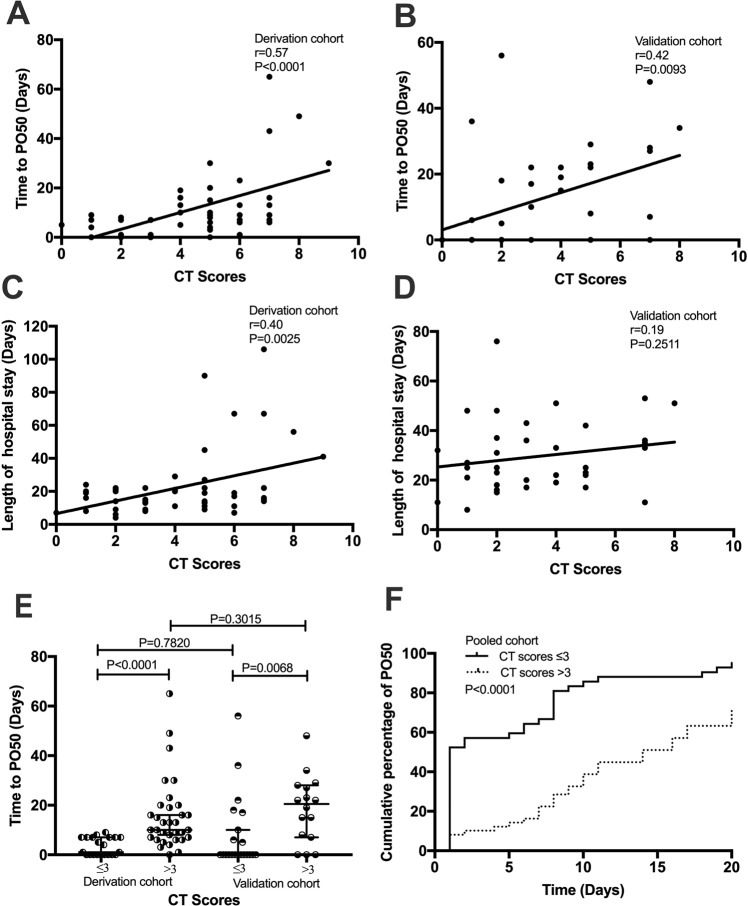
CT scores predict GI functional outcome. CT score for VPO was positively correlated with patients’ time to PO50 (**A**,**B**). CT score for VPO was lesser extent correlated with the length of hospital stay (**C**,**D**). Comparisons of time to PO50 in patients with different CT scores (**E**). Kaplan-Meier curve presenting the cumulative percentage of PO50 with different CT scores over the follow-up period in pooled cohort (**F**). Comparison was performed using log-rank (Mantel-Cox) test.

CT score for SLE GI involvement was positively correlated with patients’ time to PO50 (r = 0.57, p < 0.0001 in the derivation cohort; r = 0.42, p = 0.0093 in the validation cohort, Fig. 3A,B), and to a lesser extent correlated with the length of hospital stay (r = 0.40, p = 0.0025 in the derivation cohort; r = 0.19, p = 0.25 in the validation cohort, Fig. 3C,D). Patients with a CT score ≤3 (low CT score group) tended to have a more rapid reversible course with a median time to PO50 of 1 (IQR: 0–7) and 0 (IQR: 0–13.5) days in the derivation and validation cohorts, respectively; whereas patients with a CT score>3 (high CT score group) had a significantly prolonged recovery with a median time to PO50 of 10 (IQR: 6.5–19.5; p < 0.0001) and 20.5 (IQR: 7.3–27.8; p = 0.0068) days, respectively (Fig. 3E). Kaplan-Meier curves also demonstrated a more rapid dietary recovery in the low CT score patients of SLE GI involvement (p < 0.0001 in the pooled cohort, Fig. 3F).

Likewise, more total parenteral nutrition (TPN) was prescribed in the high CT score group (90.1% vs 52.4%, p = 0.024 in the derivation cohort; 81.3% vs 38.1%, p = 0.018 in the validation cohort). Moreover, the proportions of patients who underwent TPN ≥ 7 days were also much higher in the patients with a high CT score (60.6% vs 4.8%, p < 0.0001 in the derivation cohort; 62.5% vs 23.8%, p = 0.021 in the validation cohort; 61.2% vs 14.3%, p < 0.0001 in pooled cohort, Table 3).

**Table 3:** Comparisons of clinical and laboratory indicators between the two groups of different CT scores.

	Derivation cohort			Validation cohort		
	Low CT score group (n = 21)	High CT score group (n = 33)	p value	Low CT score group (n = 21)	High CT score group (n = 16)	p value
CT scores	2 (1–3)	5 (5–7)	<0.0001	2 (1–2)	5 (4.3–7)	<0.0001
Time of PO50, d	1 (0–7)	10 (6.5–19.5)	<0.0001	0 (0–13.5)	20.5 (7.3–27.8)	0.0068
Length of hospital stay, d	14 (8.5–20)	20 (13.5–29)	0.010	25 (17.5–36.5)	29 (22–40.5)	ns
Age, y	33 (26.5–41)	34 (26.5–42)	ns	31.5 ± 8.6	32.7 ± 10.1	ns
Female, n(%)	21 (100)	31 (93.9)	ns	20 (95.2)	15 (96.2)	ns
Disease duration, m	30 (12–57)	18 (6–42)	ns	11 (1–87)	11.5 (2.5–78)	ns
Duration of GI symptoms to CT, w	4 (0.5–14.5)	1.5 (0.5–3.5)	ns	5 (3–7.5)	7.5 (2.3–20.3)	ns
SLEDAI score	7 (4–9)	8 (6–12.5)	ns	6 (3.5–16)	7 (3.25–12.75)	ns
Albumin, g/L	32.1 ± 5.2	29.9 ± 6.0	ns	30.9 ± 8.0	30.3 ± 5.9	ns
Serum amylase, U/L	79 (67–93.5)	80 (61–103.5)	ns	72.5 (51.3–277.8)	88 (69–120.5)	ns
IgG, g/L	12.9 (10.7–16.1)	13.4 (10.5–17.2)	ns	18.3 ± 8.6	13.2 ± 4.8	ns
C3, g/L	0.59 ± 0.23	0.42 ± 0.16	0.0027	0.41 (0.37–0.49)	0.50 (0.29–0.67)	ns
Anti-dsDNA antibody, n(%)	14 (71.4)	22 (67.7)	ns	12 (57.1)	7 (43.8)	ns
Active LN, n(%)	7 (33.3)	14 (42.4)	ns	6 (28.6)	8 (50.0)	ns
NPSLE, n(%)	0 (0.0)	2 (6.1)	ns	3 (14.3)	1 (6.3)	ns
Dosage of GC > 2 mg/kg/d, n(%)	9 (42.9)	24 (72.7)	0.028	9 (42.9)	12 (57.1)	ns
TPN, n(%)	11 (52.4)	30 (90.1)	0.0024	8 (38.1)	13 (81.3)	0.018
TPN ≥ 7d, n(%)	1 (4.8)	20 (60.6)	<0.0001	5 (23.8)	10 (62.5)	0.023

The clinical manifestations and laboratory findings were compared between low CT score (≤3) and high CT score (>3) groups (Table 3). Patients in the high CT score group had lower serum C3 levels in the derivation cohort, but not in the validation cohort. No other clinical parameters displayed consistent differences between the two groups in both cohorts.

Data are presented as median with interquartile ranges (Q1-Q3) if the distribution was skewed and otherwise expressed as mean ± SD for continuous variables and number (frequency) (%) for categorical variables.

CT, computed tomography; PO50, intake 50% ideal calories PO; SLEDAI, SLE disease activity index; IgG, immunoglobulin G; LN, lupus nephritis; NPSLE, neuropsychiatric SLE; GC, glucocorticoid (prednisone); TPN, total parenteral nutrition.

## Discussion

Our two independent cohorts of SLE GI involvement, presented with abdominal pain, nausea/vomiting and diarrhea, attributed to active SLE, excluding other causes, were representative patients with this condition. Moreover, according to the CT imaging findings, half cases had GI tract involvement alone, whereas most of the rest also had extra-GI compartment involvement. In addition, the jejunum, ileum, and colon were the most commonly affected bowel segments. Among the extra-GI involvements, the urinary tract and biliary tract were the most frequently affected compartments. These clinical patterns are consistent with previous reports^15–18^.

The typical image feature of SLE GI involvement with diffuse intestinal wall thickening in line with GI dysfunction, which makes the term intestinal pseudo-obstruction (IPO)^4–7^ more appropriate for the clinical scenario. Conversely, the terminology of mesenteric vasculitis is getting less popularity owing to it rarely had pathology support. Additionally, another signature of this condition is that it is not anatomically restricted to the GI tract, but rather, with multi-compartment extra-GI visceral involvement, including ureterohydronephrosis and interstitial cystitis, cholecystitis, biliary tract dilatation, pancreatic duct dilatation and pancreatitis^5,9,19^. The image pattern implies a common pathophysiology, i.e., an immune-mediated diffuse smooth muscle dysfunction probably linked to aberrant innervations with or without a vasculitic or vasculopathic background^7^. We thus proposed to introduce ‘visceral pseudo-obstruction’ (VPO) as the new terminology to encompass those SLE GI involvement and beyond, in order to capture the characteristics of such conditions and facilitate further research.

**Figure 4:**
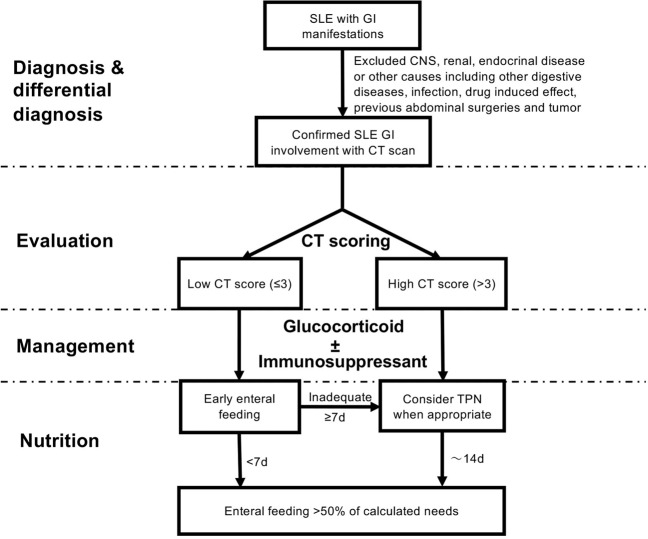
A flow chart of SLE-VPO evaluation and management based on CT scoring system^32^.

Taken the extent of intestinal wall thickening and multi-compartment involvement into considerations, our composite CT image-based scoring system provides a new assessment tool for SLE GI involvement or VPO. The scoring system incorporated both anatomical distribution and intestinal wall thickness, which are known to be relevant to GI dysfunction^18–24^. We chose the time to PO50 as the parameter to reflect the time to GI function recovery^25^. The CT score was significantly correlated with this outcome measurement. The clinical implication of the CT scores for SLE-VPO is of importance. As opposed to GI dysfunction secondary to inflammatory bowel disease, surgery and pancreatitis, where their own dietary guidelines are available^26–29^; the nutritional protocol for SLE GI involvement is merely empirical. Therefore, the CT scoring system may be helpful to predict the GI function recovery and shape the nutritional approach for SLE-VPO. According to our data, patients with a high CT score (>3) had a significantly prolonged recovery with a median time to PO50 of 13 days, indicating that these patients probably need TPN as supportive care. Indeed, more patients were prescribed TPN in the high CT score group in both cohorts. On the other hand, SLE-VPO patients with a CT score ≤3 tend to have a more rapid reversible course with a median time to PO50 of 0 day in pooled cohort. Hence, early enteral feeding should be attempted for such patients. A tentative flow chart based on our CT scoring system was provided to outline the timing of dietary recovery and TPN use for SLE-VPO (Fig. 4).

The VPO terminology and the CT scoring system might be useful for clinical evaluation of SLE GI involvement; however, there are some limitations for the current study. First, some rarer clinical entities in SLE, such as protein-losing enteropathy, mesenteric thrombosis (with or without anti-phospholipid syndrome), inflammatory bowel disease-like conditions, and isolated acute pancreatitis, were not enclosed in the study. Second, the CT scoring is more or less investigator-dependent, and artificial factors may have certain impact. For examples, poor intestinal preparation and obstruction associated with extreme dilatation of GI segments may cause measurement errors of the intestinal wall thickness. Moreover, the timings of patients’ CT scan are also variable that may have influenced the scoring. And all the patients were admitted to hospital, which suggested that our cohorts may be biased with more severe disease. Third, the retrospective design limited the interpretation of nutritional approach. Therefore, a prospective, multi-centered study is mandatory not only to further validate and optimize the scoring system, but also to provide reliable clinical evidence to guide appropriate management. Last but not least, the radiation exposure of CT scan should always bear in mind. The application of magnetic resonance enterography (MRE) is very promising, particularly in inflammatory bowel disease evaluation. After overcoming certain technical barriers, MRE should be evaluated in the context of SLE GI involvement in the future.

In conclusion, the CT scoring system and the proposed terminology of VPO may facilitate the assessment and individualized treatment for SLE patients with GI involvement. It may also be helpful in terms of future clinical trial design and in-depth mechanistic research for this unique complication of lupus.

## Methods

Two cohorts of patients with SLE GI involvement were retrospectively included from Shanghai Renji Hospital (derivation cohort) and Peking Union Medical College Hospital (validation cohort). All patients with SLE GI involvement admitted to hospital between September 2013 and May 2018 fulfilled the following inclusion criteria: 1) the American College of Rheumatology (ACR) 1997 revised classification criteria for SLE; 2) with GI manifestations, such as abdominal pain, nausea/vomiting, and diarrhea, that were attributed to SLE activity, which were retrospectively reviewed and confirmed by investigators taking into account all other secondary factors (see exclusion criteria) and immunosuppressive treatment response; 3) each patient had took a baseline abdominal CT scan with intravenous and oral contracts. Exclusion criteria: (1) patients with other digestive diseases, such as inflammatory bowel disease, irritable bowel syndrome, and celiac disease; (2) GI manifestations caused by central nervous system (CNS), renal and endocrinal disease, infection, drug-induced effect or tumor; (3) history of previous abdominal surgeries.

Patients’ demographic data, clinical characteristics, treatments and outcomes were documented. The time to GI functional recovery was the primary outcome measurement, defined as the time to tolerable PO50 without refeeding symptoms^30,31^, which was evaluated by a nutritionist blinded to the CT scan data. Secondary outcome was the length of stay in hospital. Written informed consent was obtained from each patient. All procedures performed in the study involving human participants were in accordance with the ethical standards of the Helsinki Declaration and its later amendments or comparable ethical standards. The study was approved by the Ethics Committee of Renji Hospital (IRB # 2017–041).

### CT imaging analysis

All patients underwent abdominal CT scan at the baseline using a Somatom Force scanner (Siemens Healthcare). Scans were obtained with 1.0–1.5-mm-thick sections and 1.0–1.5-mm intervals. All patients took sufficient water (1000–2000mL depend on tolerance) as a negative contrast medium for GI preparation within 30 min prior to CT-scanning. Intravenous contrasts (iopamidol) was administered during the procedure.

Abdominal CT scans were retrospectively reviewed by two experienced investigators. The investigators in charge of CT scoring were blinded to the symptoms and outcomes of patients. SLE patients with GI manifestations due to other secondary reasons (exclusion criteria) whose abdominal CT scans were used as a control and training group for calibration the cutoff of the intestinal wall thickness (Supplemental Fig. 1). CT scan findings were documented according to anatomic distribution. The most prominent thickness of each GI segment (duodenum, jejunum, ileum, colon) was measured; and the involvements of other abdominal compartments, including urinary tract, gall bladder and biliary tract, pancreas and pancreatic duct, were recorded.

### Statistical analysis

The software package SPSS 20.0 (IBM Armonk, NY, USA) was used to perform the statistical analyses. Continuous variables were expressed as median with interquartile ranges (IQR) if the distribution was skewed and otherwise expressed as means ± standard deviations (SD). Categorical data were expressed as absolute values and percentages. Independent sample t test, nonparametric Mann-Whitney U test, Chi-squared tests, Fisher’s exact test, or linear regression were applied as indicated. The consistency of the readout between investigators was evaluated by Kappa test. Cumulative percentage of patients reaching PO50 was calculated by Kaplan–Meier analysis, and comparisons were made by the log-rank test. A p value of <0.05 was considered statistically significant.

## Supplementary information

Supplementary material.
