# Supplementary material for: Systemic lupus erythematosus gastrointestinal involvement: a computed tomography-based assessment
Source: Sci Rep. 2020 Apr 14;10:6400. doi: 10.1038/s41598-020-63476-9 (PMC7156738; doi:10.1038/s41598-020-63476-9)
Supplement: Supplementary file 1 — Supplementary material. [file 41598_2020_63476_MOESM1_ESM.pdf]

Supplementary Material

Systemic lupus erythematosus gastrointestinal involvement: a computed tomography-based assessment

Zhiwei Chen[1*], Jiaxin Zhou[2*], Jiaoyu Li[1], Yiquan Zhou[3], Xiaodong Wang[1], Ting Li[1], Liyang Gu[1], Fangfang Sun[1], Wanlong Wu[1], Wenwen Xu[1], Shuhui Sun[1], Jie Chen[1], Jiajie Li[1], Liangjing Lu[1], Wen Zhang[2], Yan Zhao[2], Shuang Ye[1]

1 Department of Rheumatology, Renji Hospital South Campus, Shanghai Jiaotong University School of Medicine, Shanghai, 201112, China

2 Department of Rheumatology, Peking Union Medical College Hospital, Peking Union Medical College and Chinese Academy of Medical Sciences, Key Laboratory of Rheumatology and Clinical Immunology, Ministry of Education, Beijing, 100730, China

3 Department of Clinical Nutrition, Renji Hospital, Shanghai Jiaotong University School of Medicine, Shanghai, 200127, China

*These authors contributed equally to this work.

Correspondence and requests for materials should be addressed to Shuang Ye (email: ye_shuang2000@163.com) or Yan Zhao (email: zhaoyan_pumch2002@aliyun.com)

Supplemental Part I：

Supplemental Figure 1 Flow chart of patients' enroll and comparisons in the derivation cohort

Supplemental Part II

Supplemental Table 1 Linear regression between CT scores and time to PO50 by different CT score systems in derivation cohort

Supplemental Part I

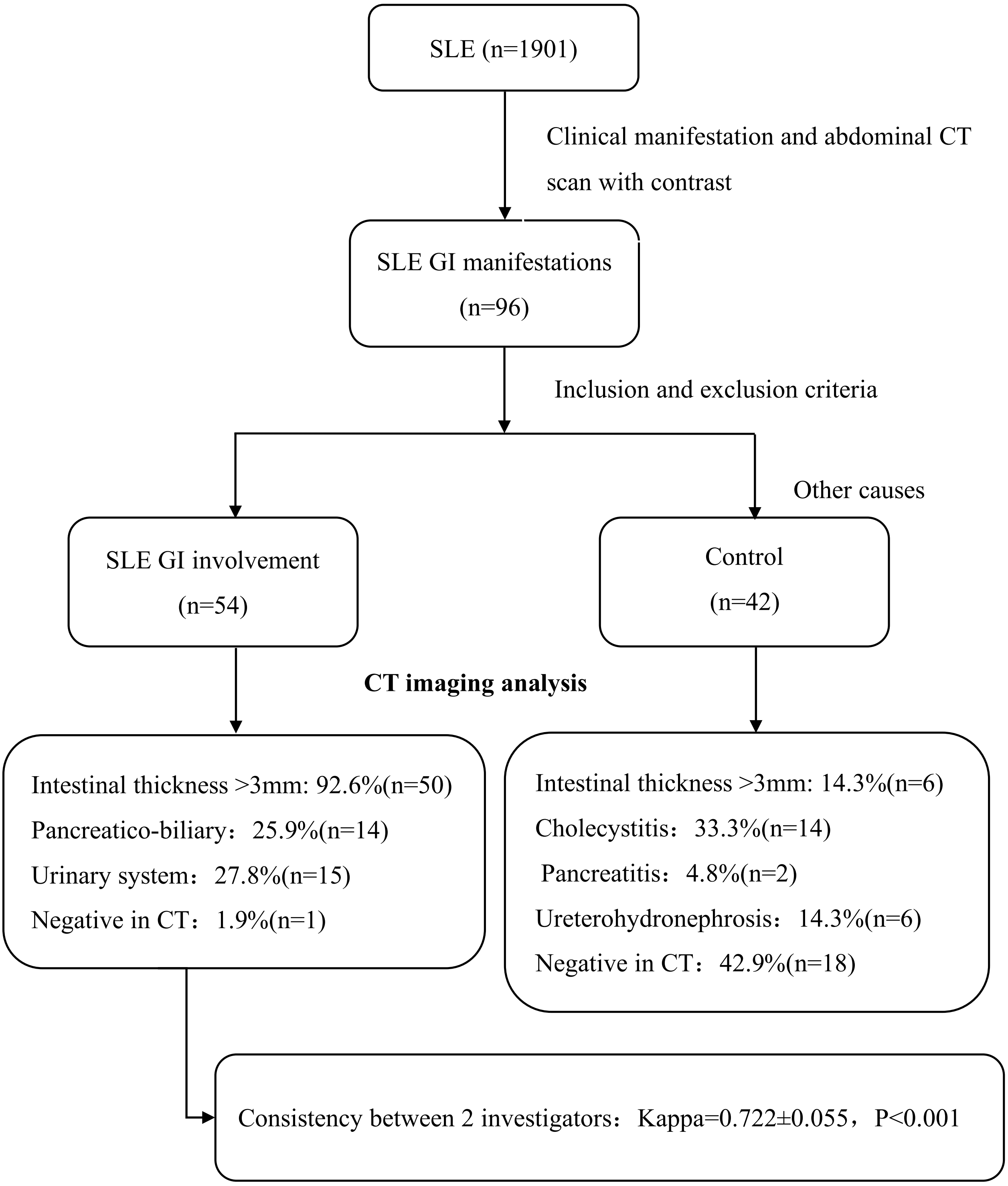

Supplemental Figure 1 Flow chart of patients' enroll and comparisons in the derivation cohort

Supplemental Prat II

Supplemental Table 1 Linear regression between CT scores and time to PO50 by different CT score systems in derivation cohort (n=54)

CT score system	GI		Extra-GI compartments**	r	p value
	Segments*	Thickness#			
Combo 1	4	Yes	4	0.57	<0.0001
Combo 2	4	Yes	2	0.55	<0.0001
Combo 3	4	Yes	0	0.54	<0.0001
Combo 4	4	No	4	0.45	0.0006
Combo 5	4	No	2	0.44	0.0008
Combo 6	4	No	0	0.42	0.0014
Combo 7	2	Yes	4	0.45	0.0006
Combo 8	2	Yes	2	0.50	0.0001
Combo 9	2	Yes	0	0.47	0.0003
Combo 10	2	No	4	0.34	0.0127
Combo 11	2	No	2	0.34	0.0112
Combo 12	2	No	0	0.36	0.0079

*GI segments: 2 parts including small intestine and colon; 4 parts including duodenum, jejunum, ileum and colon.

#When thickness of bowel wall ≥8.0mm, whether to get a bonus 1 score of each segment.

**Extra-GI compartments: 2 parts including pancreatico-biliary system and urinary system; 4 parts including biliary tract, pancreas, renal pelvis/ureter and bladder.
